# Early life programming by diet can play a role in risk reduction of otitis in dogs

**DOI:** 10.3389/fvets.2023.1186131

**Published:** 2023-11-06

**Authors:** Manal B. M. Hemida, Kristiina A. Vuori, Nona C. Borgström, Robin Moore, Sarah Rosendahl, Johanna Anturaniemi, Alessandra Estrela-Lima, Anna Hielm-Björkman

**Affiliations:** ^1^Department of Equine and Small Animal Medicine, Faculty of Veterinary Medicine, University of Helsinki, Helsinki, Finland; ^2^Department of Nutrition and Clinical Nutrition, Faculty of Veterinary Medicine, Beni-Suef University, Beni-Suef, Egypt; ^3^Department of Veterinary Anatomy, Pathology and Clinics, School of Veterinary Medicine and Zootechny, Federal University of Bahia, Salvador, Brazil

**Keywords:** DogRisk, nutrition, ear, inflammation, canine, early, programming, DOHaD

## Abstract

**Introduction:**

Otitis in dogs is often chronic while local treatment primarily consists of flushing, antibiotics, and/or antifungals. We were interested in finding early life variables that associate with otitis later in life, preferably some that could be modified.

**Methods:**

A cross-sectional hypothesis-driven study with longitudinal data was performed to search for associations between pre- and postnatal exposures, and the incidence of owner-reported otitis in dogs at over 1 year of age. The multivariate logistic regression analysis study included data from 3,064 dogs and explored 26 different early life variables at four early life stages: prenatal, neonatal, postnatal, and puppyhood. We compared two feeding patterns, a non-processed meat-based diet (NPMD, raw) and an ultra-processed carbohydrate-based diet (UPCD, dry).

**Results:**

We report that eating a NPMD diet significantly decreased the risk of otitis later in life, while eating a UPCD diet significantly increased the risk. This was seen in different life stages of mother or puppy: The maternal diet during pregnancy (*p*=0.011) and the puppies’ diet from 2 to 6 months of age (*p*=0.019) were both significantly associated with otitis incidence later in life, whereas the puppies’ first solid diet, was associated in the same way, but did not reach significance (*p*=0.072). Also, analyzing food ratios showed that when puppies were consuming >25% of their food as NPMD it significantly decreased their incidence of otitis later in life, while a ratio of >75% UPCD in their diet significantly increased their risk of otitis. Also, if the dog was born in the current family, was exposed to sunlight for more than 1 hour daily, and was raised on a dirt floor during puppyhood, there was a lower risk of otitis development later in life.

**Discussion:**

The findings only suggest causality, and further studies are required. However, we propose that veterinarians, breeders, and owners can impact otitis risk by modifying factors such as diet and environment.

## Introduction

Otitis in dogs is a frequent inflammatory disease, seldom diagnosed as a primary disease, and it may affect any of the ear parts, either external ear canal (otitis externa) (1), middle ear (otitis media), and/or the inner ear (otitis interna) (2). Otitis in dogs is associated with alterations in the ear microbiota (3). The prevalence of the disease has been estimated in Europe to range from 8.7 to 20% (4–6). The etiology of otitis in dogs is multifactorial where various factors have been contributed to its pathogenesis (7–9). Recently its etiology has been classified into Primary, Secondary, Predisposing, and Perpetuating causative factors (2) which are abbreviated into the PSPP system (10). The main reasons for otitis development in dogs are allergies, atopic dermatitis (AD), food hypersensitivity, allergen contact, otoacariasis, and autoimmune and endocrine diseases (2, 9, 11–13), which are perpetuated by secondary yeast and/or bacterial infections (8). The age of onset of otitis in dogs is highly variable, since it differs based on the underlying primary cause (11).

The genetic nature and breed predisposition to develop otitis have been reported in specific dog breeds [(14); Supplementary Table S1]. A higher incidence of otitis is often seen within breeds with pendulous/long ears (7), although the reduced incidence of otitis within some breeds with pendulous ears indicates that congenital factors alone do not determine disease incidence. The Developmental Origin of Health and Disease (DOHaD) hypothesis suggests that early life exposures, especially early diet, during the critical developmental periods; pre- and postnatal, modulate developmental programming via epigenetics and the establishment of the early microbiome, thereby stimulating the immune system and determining the dog’s susceptibility to diseases later in life, including allergies and autoimmune diseases (15, 16).

There are numerous potential mechanisms involved in early life programming by the diet, among them genetics and epigenetics, microbiome establishment, and fetal organogenesis (15–18). Previously we investigated the importance of early life nutritional and environmental exposures in several studies. We have looked at time periods starting from prenatal life till one and a half years old and analyzed the risk of canine atopic dermatitis and inflammatory bowel disease/canine chronic enteropathy in dogs (19–22). Thus, identification and elimination of the most primary causes of otitis such as allergies, canine atopic dermatitis, and other primary etiologies early in life, through early life programming of the individual’s immune system, are possible and highly practical preventive approaches for reducing the risk of otitis incidence in dogs. The current study aimed to investigate the role of the early life modifiable and non-modifiable exposures on later onset of otitis in dogs.

## Materials and methods

### Data source and outcome measure

We scrutinized data from the validated (23) owner-reported DogRisk food frequency questionnaire (FFQ) with a cross-sectional and longitudinal design (available in Finnish at: http://bit.ly/427aGBa). The FFQ was developed at the University of Helsinki, Finland and was available for dog owners online from 2009 to 2021. The questionnaire was widely disseminated to Finnish dog owners via several professional, public, and social platforms as described in a previously published study (24). As an epidemiological tool of preventive medicine, the FFQ was screening potential causes of non-communicable diseases in Finnish dogs. The FFQ included different categories of questions regarding the dogs’ disease diagnoses and life-long exposures, including nutrition of the dog and its dam, environmental indoor and outdoor exposures, history of maternal diseases, breed, sex, age, coat color, etc. More information on the FFQ has been presented in prior research (19–22, 24). The FFQ was approved by the ethical board of Viikki campus, University of Helsinki (29.4.2016).

**Figure 1:**
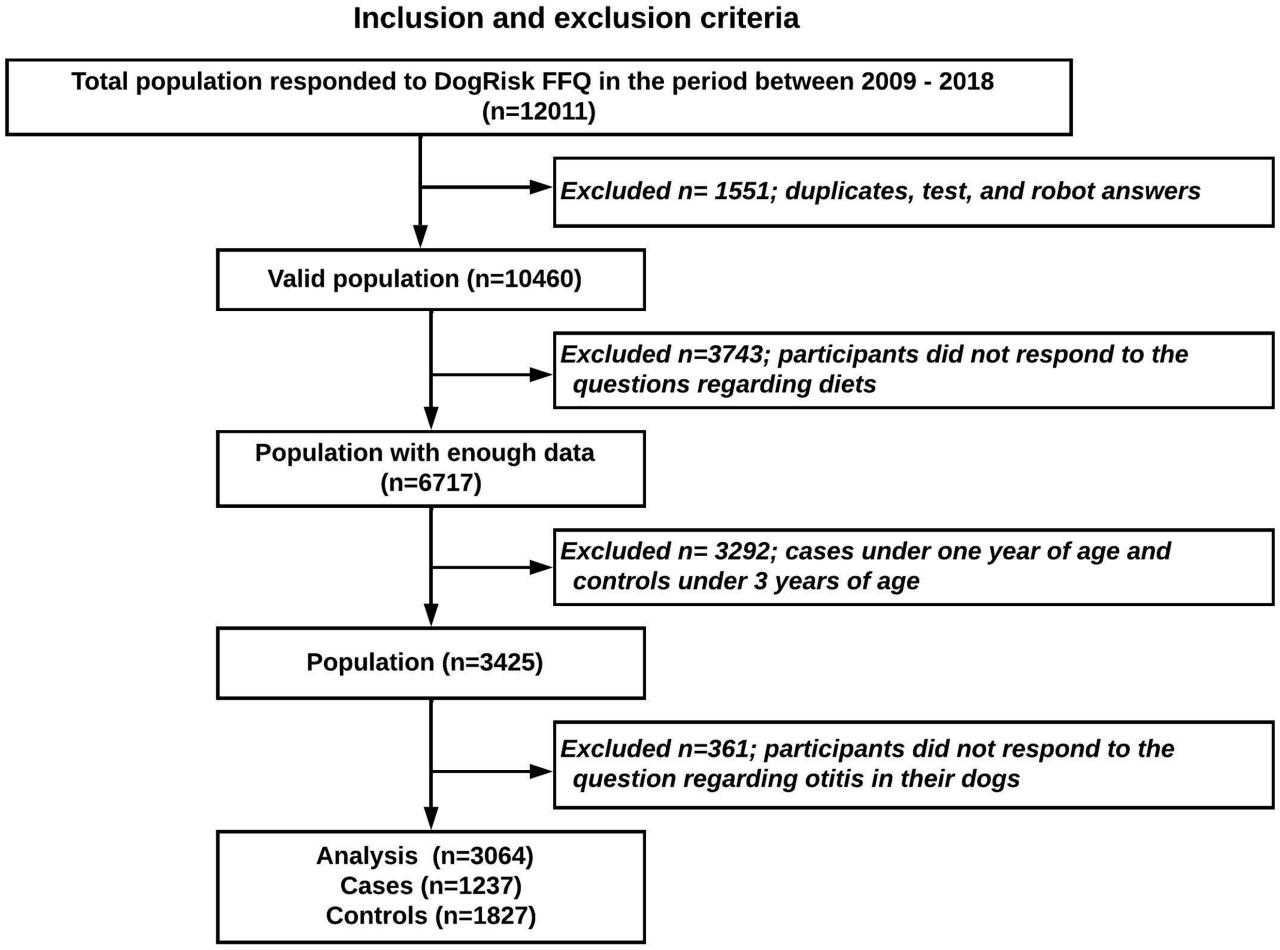
Study population flowchart.

The dichotomized (yes/no) outcome question to the owner was: Has your dog suffered from otitis/inflammation of the ear? From a total of 10,4601 participants that answered the FFQ between 2009 and 2018, a study sample of 3,064 dogs (1,237 cases and 1,827 controls) was analyzed. To avoid reverse causality all cases under 1 year of age and all controls under 3 years of age were excluded (shown in the study flowchart, Figure 1).

### Study setting, design, and early-life tested variables

**Figure 2:**
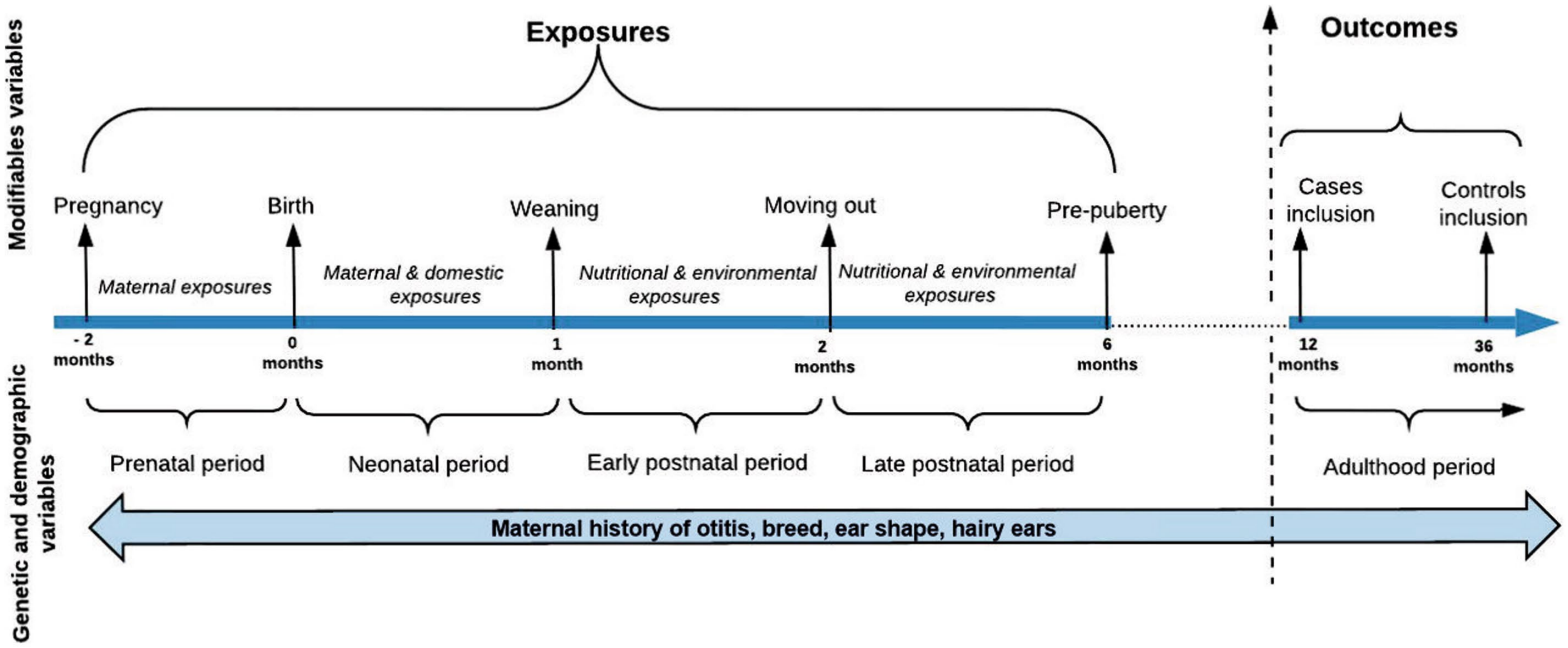
Study timeline. The diagram modified from Hemida et al. (19).

A cross-sectional epidemiological study with longitudinal data was performed to look for the associations between early life exposures and the development of otitis in dogs at over 1 year of age. The study included one dichotomous dependent variable and 26 dichotomous and categorical independent covariates (Supplementary Table S2). Modifiable and non-modifiable exposures from the four early periods of the dog’s life were selected based on previous research and classified into five groups of analysis according to their time of exposure: group I included the prenatal non-modifiable exposures, group II included the prenatal modifiable exposures, group III included the neonatal modifiable exposures, group IV included the early postnatal modifiable exposures, and group V included the late postnatal modifiable exposures (Table 1, Figure 2). The early life variables included in the analysis were heterogeneous, obtained from different categories of exposure such as genetic, hereditary, demographic, dietary, environmental, domestic, and immune-related (Supplementary Table S2). Two breed-related variables were created based on the breed information available at the Federation Cynologique Internationale (FCI)^1^: (i) the dog’s ear shape (erect, semi-erect, or dropped ears) and (ii) the presence of hair in the ears (“hairy/pilose ears” or “non-hairy or pilose ears”). Based on that and breed disposition literature mentioned in Supplementary Table S1, the DogRisk FFQ breeds have been classified into two groups: otitis prone and otitis non-prone breeds. The methods used for data preparation have been described previously (19). Three open answers questions were asking about the maternal diets during pregnancy and lactation, and the puppy’s first solid diet. Based on the answers, the dams and puppies were divided into either ultra-processed carbohydrate-based diet (UPCD) or non-processed meat-based diet (NPMD) eating dogs. Dogs that were on other diets were not included in this study. The puppies’ diets from 2 to 6 months of age were extracted from a diet ratio question where the owner could choose the consumed % from four kinds of diets; UPCD, NPMD, processed wet, and home-cooked diets. From the latter we only analyzed two extreme groups; the dogs that had either been eating over 80% of UPCD or over 20% of NPMD in their diets. These percentages were chosen based on previous research (21). Additionally, we examined two ratio scales to evaluate the prevalence of otitis among the study sample when consuming different ratios (0%, 1–25%, 26–50%, 51–75%, 76–100%) of the two, NPMD and UPCD, feeding patterns.

**Table 1:** Associations between pre-, neo-, early post-, and late postnatal period variables and otitis disease based on univariate logistic regression analyses.

Covariates	Total population for analysis (*n* = 3,064)		Crude effect estimates	
	Included dogs (*n*)	Missing dogs (*n*)	cOR (95% CI)	*p*-value
I. Prenatal period				
Genetic and demographic factors; non-modifiable				
*1. Maternal history of otitis*	1,295	1,769		
Mothers without otitis vs. mothers with otitis			0.114 (0.082–0.158)	<0.001*
Mothers with otitis vs. mothers without otitis			8.760 (6.312–12.157)	
*2. Dog’s AASS*	2,936	128		
Dogs without AASS vs. dogs with AASS			0.264 (0.219–0.317)	<0.001*
Dogs with AASS vs. dogs without AASS			3.795 (3.156–4.564)	
3. Dog’s ear shape (Ref. Erect ears)	2,394	670		
Semi-erect vs. erect ears			1.343 (1.039–1.736)	0.024*
Dropped vs. erect ears			2.668 (2.208–3.225)	<0.001*
*4. Hairy/pilose ears*	2,394	670		
Hairy/pilose vs. non-hairy/pilose ears			1.301 (1.043–1.622)	0.020*
Non-hairy/pilose vs. hairy/pilose ears			0.769 (0.616–0.959)	
***5. Dog breed***	2,340	724		
Otitis non-prone vs. prone breed			0.400 (0.337–0.474)	<0.001*
Otitis prone vs. non-prone breed			2.503 (2.112–2.967)	
*6. Dog color*	2,875	189		
<50% white colored coat vs. >50%			0.927 (0.770–1.116)	0.425
>50% white colored coat vs. <50%			1.078 (0.896–1.298)	
7. Dog sex	2,980	84		
Female vs. male			0.817 (0.705–0.946)	0.007*
Male vs. female			1.224 (1.057–1.418)	
Maternal factors; modifiable				
*8. Mother’s diet during pregnancy*	1,896	1,168		
NPMD vs. UPCD			0.607 (0.417–0.882)	0.009*
UPCD vs. NPMD			1.648 (1.133–2.396)	
*9. Was the mother dewormed during pregnancy?*	1,878	1,186		
Yes vs. no			1.128 (0.630–2.019)	0.685
No vs. yes			0.886 (0.495–1.586)	
*10. Was mother vaccinated during pregnancy?*	1,082	1,982		
Yes vs. no			1.143 (0.893–1.463)	0.289
No vs. yes			0.875 (0.683–1.120)	
II. Neonatal period (0–1 month); modifiable factors				
*11. Mother’s diet during lactation*	1,798	1,266		
NPMD vs. UPCD			0.760 (0.525–1.099)	**0.144**
UPCD vs. NPMD			1.317 (0.910–1.905)	
*12. Was the dog born in the same family?*	3,064	0		
Yes vs. no			0.431 (0.315–0.590)	<0.001*
No vs. yes			2.321 (1.695–3.177)	
*13. Season of birth (Ref. Autumn)*	3,016	48		
Winter vs. autumn			1.175 (0.945–1.461)	**0.147**
Spring vs. autumn			0.998 (0.810–1.231)	0.988
Summer vs. autumn			0.929 (0.741–1.164)	0.520
III. Early postnatal period (1–2 months); modifiable factors				
*14. Puppy’s first solid diet*	1,853	1,211		
NPMD vs. UPCD			0.714 (0.496–1.028)	**0.070**
UPCD vs. NPMD			1.400 (0.972–2.016)	
*15. Frequency of outdoor activity (Ref. Not at all)*	2,318	746		
Many times / day vs. not at all			0.733 (0.542–0.991)	0.043*
Once/day vs. not at all			0.913 (0.645–1.292)	0.607
A few times / week vs. not at all			1.058 (0.735–1.523)	0.763
A few times / month vs. not at all			1.334 (0.839–2.123)	0.224
*16. Sunlight exposure, hours / day*	1,672	1,392		
≥ 1 vs. not at all			0.697 (0.572–0.849)	0.001*
Not at all vs. ≥ 1			1.435 (1.178–1.749)	
*17. Type of flooring*	2,420	644		
Dirt / lawn vs. non-dirt / lawn floor			0.735 (0.526–1.026)	**0.07**
Non-dirt / lawn vs. dirt / lawn floor			1–361 (0.975–1.901)	
*18. Body condition Score*	2,570	494		
Normal weight vs. underweight			0.819 (0.625–1.072)	**0.145**
Underweight vs. normal weight			1.222 (0.933–1.600)	
Normal weight vs. overweight			1.045 (0.833–1.311)	0.703
Overweight vs. normal weight			0.957 (0.762–1.201)	
Overweight vs. underweight			0.783 (0.564–1.088)	**0.145**
Underweight vs. over weight			1.277 (0.919–1.773)	
IV. Late postnatal period (2–6 months); modifiable factors				
*19. Puppy diet*	1,471	1,593		
NPMD vs. UPCD			0.627 (0.431–0.911)	0.014*
UPCD vs. NPMD			1.595 (1.098–2.318)	
*20. Outdoor activity, hours / day (Ref. < 0.5)*	2,560	504		
0.5–1 vs. < 0.5			1.144 (0.664–1.971)	0.629
1–2 vs. < 0.5			1.134 (0.664–1.934)	0.646
> 2 vs. < 0.5			0.866 (0.496–1.510)	0.611
*21. Sunlight exposure, hours / day*	2,342	722		
≤ 1 vs. > 1			1.121 (0.930–1.350)	0.230
> 1 vs. ≤ 1			0.892 (0.741–1.075)	
*22. Type of flooring*	2,927	137		
Dirt / lawn vs. non-dirt / lawn floor			0.703 (0.548–0.904)	0.006*
Non-dirt/ lawn vs. dirt /lawn floor			1.421 (1.107–1.826)	
*23. Body condition score*	2,671	393		
Normal weight vs. underweight			0.864 (0.723–1.033)	**0.108**
Underweight vs. normal weight			1.157 (0.968–1.383)	
Normal weight vs. overweight			0.791 (0.578–1.083)	**0.144**
Overweight vs. normal weight			1.264 (0.923–1.730)	
Underweight vs. over weight			0.916 (0.655–1.281)	0.607
Overweight vs. underweight			1.092 (0.781–1.527)	
*24. Was the puppy vaccinated 2–4 times under 1 year of age?*	3,014	50		
Yes vs. no			1.226 (0.564–2.666)	0.607
No vs. yes			0.815 (0.375–1.773)	
*25. Was the puppy dewormed 2–10 times under 1 year of age?*	2,966	98		
Yes vs. no			2.339 (0.861–6.358)	**0.096**
No vs. yes			0.427 (0.157–1.162)	

(*n*), number of dogs; included dogs, the number of valid answers for the corresponding question; crude effect estimates, one covariate in the model each time; cOR, crude odds ratio; CI, confidence interval; AASS, atopy/allergy skin symptoms; bolded, *p* < 0.2; *, *p* < 0.05; NPMD, non-processed meat based diet; UPCD, ultra-processed carbohydrate based diet; vs., versus; Ref., reference.

### Data analysis

Data analyses were conducted using IBM SPSS Statistics 28 for Windows. Cross-tabulation was used to describe the study population and covariates’ characteristics. The prevalence of otitis within the study sample when consuming different ratios of NPMD or UPCD was also calculated using crosstabulation. Univariate logistic regression analysis was performed individually for the quantitative estimation of the association between the different covariates and the outcome by entering one independent variable at a time. The variables which had a liberal association (*p* < 0.2) with the dependent variable were selected and used for further modeling. The multivariate logistic regression analysis was done using a multi-model design to get adjusted odds ratios with 95% CI of the predictors using the backward stepwise regression method with entry and removal probability (0.1) and (0.4), respectively. Five models stretched over the four time periods (Figure 2) were analyzed, the first period included one model of non-modifiable and one of modifiable variables. Models were adjusted for age and sex at all four time points. The missing values were not imputed but handled by listwise deletion. The fitness of the models was tested and established by the Omnibus test (*p*-value < 0.05), Hosmer and Lemeshow test (*p*-value > 0.05), and Nagelkerke’s R for the largest value.

## Results

The prevalence of owner-reported otitis within the DogRisk FFQ total population after excluding the duplicates, test, and robot answers (*n* = 10,460) was 27.74%. The characteristics of the study population and the tested variables are presented in Supplementary Table S2. The study sample’s average age ± SD was 5.44 ± 2.8, in cases 5.25 ± 2.94, and in controls 5.56 ± 2.7 years old.

### Breeds’ predisposition for otitis in Finland

The otitis breed predisposition was tested among the DogRisk FFQ population’s breeds. From a total of 205 breeds (including mixed breeds), we found that 53 breeds, with a total number of 5,297 dogs, showed a significant difference between dogs with otitis and dogs without otitis in Finland. The breeds are presented in a descending order starting from breeds with the highest ratio of dogs with otitis in Table 2.

**Table 2:** Percentages of dogs with otitis within breeds showing significant difference between study cases and controls (*n* = 5,297).

Breeds^a^	Dogs with otitis within each breed, % (*n*)	Total dogs^b^/ breed, (*n*)
Chinese Shar pei	91.67 (11)	12
Weimaraner	70.00 (7)	10
Pug	68.18 (15)	22
Petit basset griffon vendeen	66.67 (8)	12
Irish glen of imaal Terrier	60.00 (6)	10
Spinone Italiano	60.00 (6)	10
Dogo Argentino	59.09 (13)	22
Welsh Springer Spaniel	58.62 (34)	58
Leonberger	57.14 (28)	49
French Bulldog	56.25 (45)	80
Bullmastiff	55.88 (19)	34
Romagna Water Dog	54.00 (27)	50
Rhodesian Ridgeback	52.94 (18)	34
Miniature poodle	52.00 (39)	75
English bulldog	51.02 (25)	49
Dalmatian	50.98 (26)	51
Irish Setter	48.65 (18)	37
Cocker spaniel	48.19 (40)	83
Border Terrier	47.27 (26)	55
Labrador Retriever	45.83 (154)	336
English Springer Spaniel	45.45 (25)	55
Spanish Water Dog	44.71 (38)	85
Newfoundland	44.07 (26)	59
Hovawart	42.98 (49)	114
Staffordshire Bull Terrier	42.08 (77)	183
Golden retriever	41.52 (93)	224
Nova Scotia Duck Tolling Retriever	39.81 (43)	108
Rottweiler	39.18 (76)	194
German Shepherd	38.08 (214)	562
Mixed breed	23.60 (283)	1,199
Finnish Lapponian Dog	18.14 (39)	215
Belgian Shepherd dog	17.16 (23)	134
Australian Shepherd	15.38 (14)	91
Lapponian Herder	15.38 (16)	104
Shetland Sheepdog (Sheltie)	10.97 (17)	155
Cardigan Welsh Corgi	10.64 (5)	47
Alaskan Malamute	8.82 (3)	34
Schipperke	8.57 (3)	35
Norwegian Elkhound Grey	8.33 (2)	24
Italian Greyhound	8.11 (3)	37
Icelandic Sheepdog	7.89 (3)	38
Pembroke Welsh Corgi	6.52 (3)	46
Siberian Husky	5.45 (3)	55
German Spitz Mittel	5.26 (2)	38
Border Collie	5.22 (7)	134
Samoyed	4.48 (3)	67
Japanese Spitz	4.17 (1)	24
Whippet	3.45 (2)	58
Australian Cattle Dog	0.00 (0)	12
Finnish Spitz	0.00 (0)	22
lancashire heeler	0.00 (0)	21
Peruvian hairless dog	0.00 (0)	14
Pharaoh Hound	0.00 (0)	10
Skye Terrier	0.00 (0)	10
Total	30.92 (1638)	5,297

a Breeds with a significant difference between dogs with otitis and dogs without otitis at *p* < 0.05.

^b^Breeds with at least 10 dogs per breed were only included in the analysis.

### Logistic regression analysis

**Figure 3:**
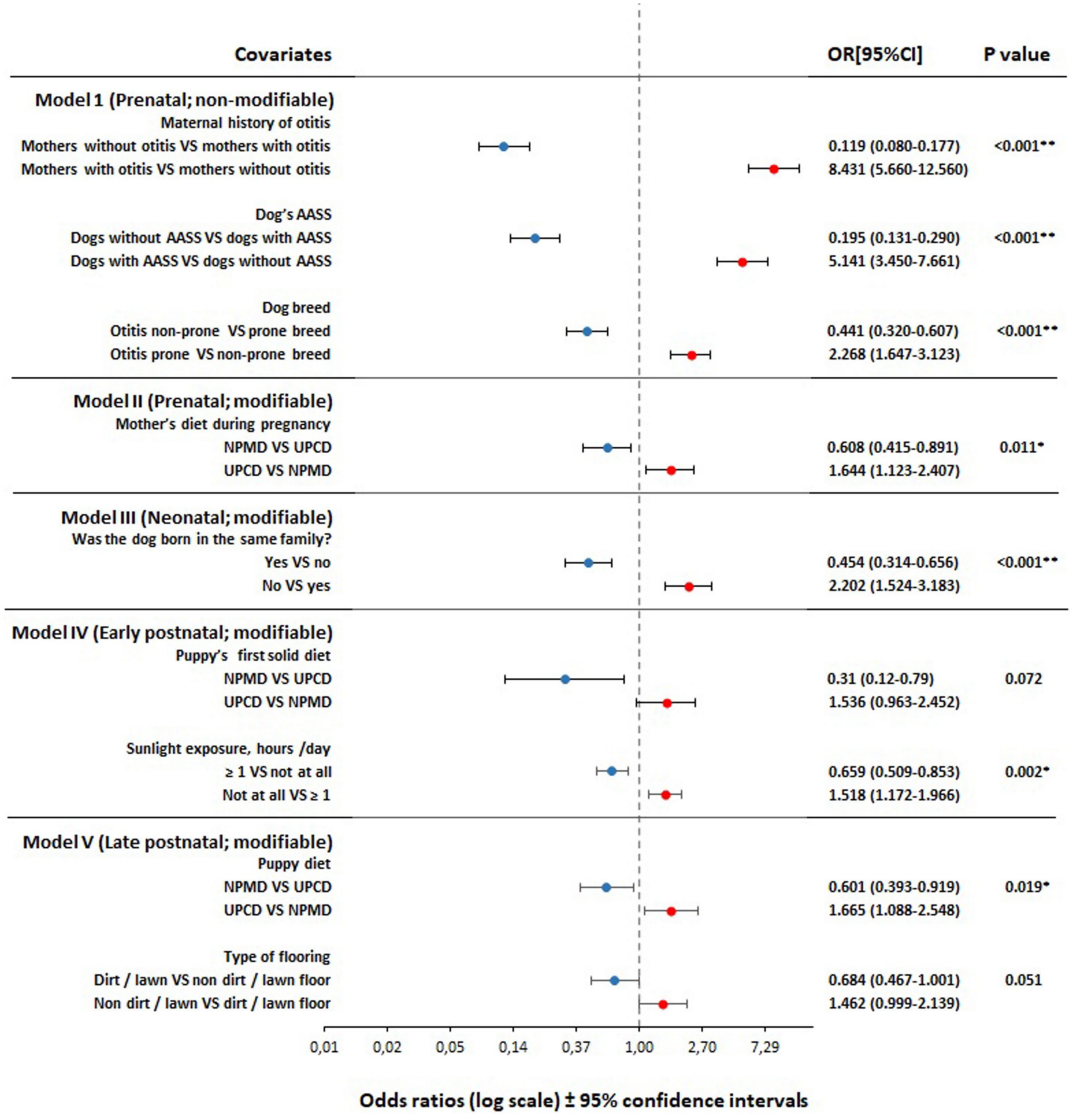
Forest plot of adjusted odds ratios for the association between pre-, neo-, early post-, and late postnatal period variables and otitis incidence in adult dogs (*n* = 3,064), based on backward stepwise multivariate logistic regression analyses. Models adjusted for age and sex. Dogs included in the analyses; Model I (*n* = 939), model II (*n* = 1,824), model III (*n* = 1,706), model IV (*n* = 1,003), and model V (*n* = 1,119). VS, versus; OR, odds ratio; CI, confidence interval; AASS, atopy/allergy skin symptoms; NPMD, non-processed meat based diet; UPCD, ultra-processed carbohydrate based diet; *, significant at *p <* 0.05; **, significant at *p <* 0.001.

Twenty variables from a total of 26 variables were found to be associated with owner-reported otitis incidence in dogs later in life with a *p* < 0.2 using the univariate logistic regression analysis, from which 12 variables were significant with *p* < 0.05 (Table 1). All twenty were forwarded for the final modeling using the multivariate analysis. From the five final models of the multivariate logistic regression, we found that seven variables were significantly associated with owner-reported otitis incidence in dogs later in life and two variables showed a tendency towards the association (*p* < 0.10 but did not reach significance at *p* < 0.05) (Figure 3).

### The effect of consuming different food ratios on the prevalence of owner-reported otitis within the DogRisk study population

**Figure 4:**
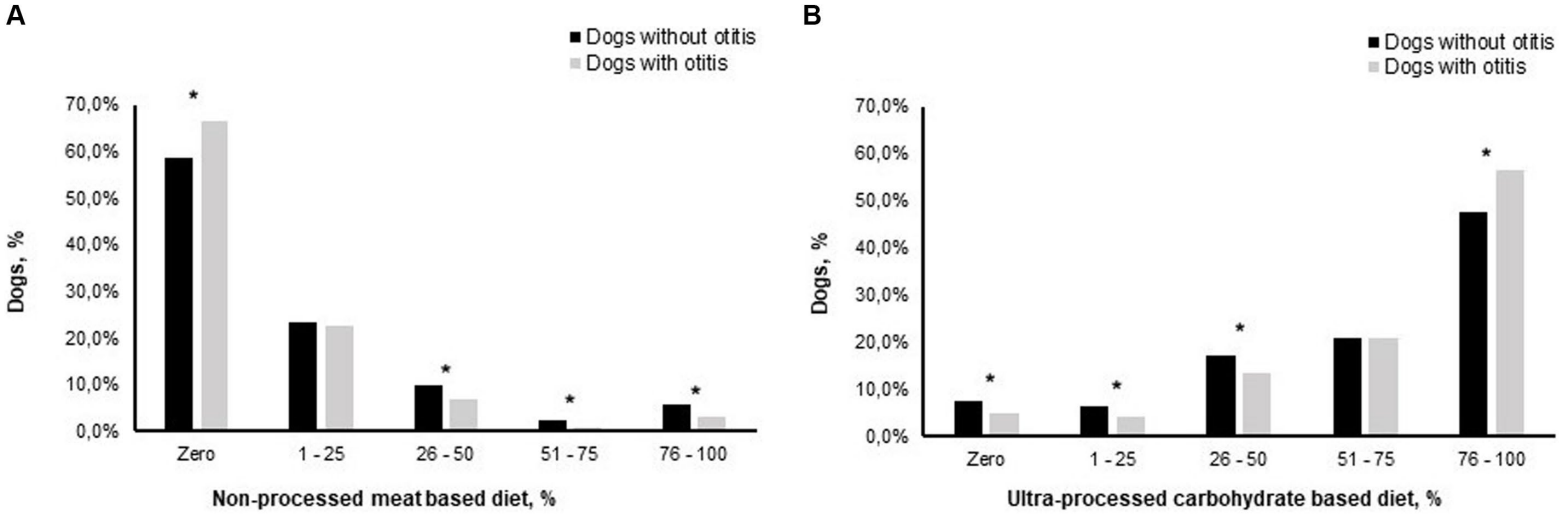
Otitis prevalence later in life is associated with feeding patterns of puppies of 2 to 6 months of age (*n* = 5,477), consuming different proportions of a non-processed meat based diet **(A)**, and an ultra-processed carbohydrate based diet **(B)**, of their total diets. *, the difference between the percentages of dogs with otitis and dogs without otitis is significant at *p* < 0.05.

From the crosstabulation analysis for determining the prevalence of owner-reported otitis in dogs when consuming different ratios from both tested feeding patterns in the current study; NPMD and UPCD, we found that the prevalence of otitis was significantly decreased when consuming >25% of their diets as NPMD. On the other hand, the prevalence of otitis was significantly increased when consuming >75% of their diets as UPCD (Figure 4).

## Discussion

### Key findings

From the perspective of preventive and supportive medicine, the main novelty of the current study is presenting the early diet as a modifiable risk factor for otitis in dogs. We found that pre- and postnatal dietary patterns have a significant impact, either negative or positive, on the development of otitis in dogs later in life (Figure 3). Our findings agree with the DOHaD hypothesis which assumes that the early life diet can program the immune system of the individual through several proposed mechanisms, providing either protection or susceptibility to diseases later in life (25–27).

### Non-modifiable risk/protective factors significantly associated with owner-reported otitis incidence in dogs at over 1 year of age

Findings from the non-modifiable genetic and background-related factors in model I showed that the maternal history of otitis remained in the model as the strongest predictor of otitis incidence in dogs (Figure 3). Maternal history of otitis was positively associated with otitis incidence in dogs later in life in the current study, where the risk increased 8.4-fold in subjects with a maternal history of otitis versus subjects without a maternal history of otitis. As far as we know, the association between the maternal history of otitis in dogs and the disease incidence in the next generation has not been explicitly investigated before. However, a recent study (7) reported the importance to exclude the parental phenotypes which are highly predisposed for otitis in designer breeding in order to reduce risk in the following generation (28). This finding is similar to our previous findings, where we found that the maternal history of canine atopic dermatitis and inflammatory bowel disease were directly associated with the same disease incidence in their offspring later in life (19, 20). From human research, the family history of otitis media and its association with otitis incidence in their children later has been suggested by several studies (29–34). This finding supports the notion that either predisposing factors (7) or genetic components (35) can be passed on to the next generation. It is also noteworthy that a newborn and up to 2 months old puppy share a common environment with the mother, including diet. This external factor could be as strong, or stronger, than a genetic or epigenetic factor and it is not possible to separate these factors in an epidemiological study of this type.

In the present study canine atopic dermatitis / atopy / allergy skin symptoms (CAD / AASS) was demonstrated to be a prominent risk factor for otitis (Figure 3). We observed a five-fold risk in dogs with AASS versus dogs without symptoms of atopy/allergy. Canine atopic dermatitis has been reported to be one of the primary causes of otitis (13). The association between CAD and otitis in dogs has been confirmed in several studies, either by the prevalence of otitis symptoms within atopic dogs (5, 12, 36–38) and/or by the concurrence of the age of the symptom’s onset (39–41). In a canine study, otitis was seen in 83% of dogs with CAD, where otitis was the initial sign in 25% of reported cases (42). Another study showed that 68% of dogs with CAD developed otitis (12). Furthermore, a study found that the age of otitis onset coincides with the age of CAD onset in dogs (39), and another study further showed that 50% of otitis cases developed at the age of 1–5 years, and 30% of otitis cases initiated before the age of 1 year (12). Moreover, a connection between the skin microbiota and the ear canal microbiota has been suggested (43). Ngo et al. (44) found that there was a difference in the composition of the ear canal microbiota between healthy and atopic dogs without symptoms of otitis, indicating that CAD is a predisposing factor for dysbiosis in the ear canal microbiota and may manifest later as otitis externa (43). Our findings agree with the wide body of literature suggesting that CAD is the underlying disease for otitis in dogs (5, 36–38, 42, 45, 46).

In the present study, dog breeds were associated significantly with otitis development. The study showed that genetically prone breeds developed otitis 2.27-fold more often than non-prone breeds. From the FFQ data, several dog breeds have been found to be prone to develop otitis (Table 2; Figure 3). The later findings are supported by other authors’ observations as shown in Supplementary Table S1. However, the wide variations in the breed predisposition of otitis are subject to several factors such as geographical location (9, 11), allergy predisposition (12, 40), lifestyle-related factors such as outdoor activity and swimming frequency (11), ear conformation (7), the presence of hair follicles within the ear canal (10), and any other predisposing factor for ear infections such as foreign bodies, excessive grooming and bathing, and systemic debilitation (7, 10). Moreover, in the current study, other predisposing factors were detected as well, such as breeds with specific ear shape or breeds with hair in the ear canal. However, although these traits were significantly associated with otitis incidence in dogs in the univariate regression analysis, they did not reach significance in the final models (Table 1).

### Modifiable risk/protective factors significantly associated with owner-reported otitis incidence in dogs at over 1 year of age

Early life diets were associated with the later development of otitis in dogs during three of the four perinatal life periods: prenatal, early postnatal, and late postnatal (Figure 3).

In the current study, consumption of NPMD by pregnant dams and by puppies during puppyhood from 2 to 6 months of age were significantly associated with a reduced risk of later otitis incidence when compared to the consumption of UPCD, while the consumption of UPCD during the same periods was significantly associated with a higher risk of otitis later in life. Although the association between the puppies’ first solid diet and otitis incidence later in life did not reach significance (*p* = 0.072), it showed a tendency towards a lower risk. Our present findings concerning early diets are consistent with our previous findings where we found that the early life diets were associated significantly with the incidence of AASS and inflammatory bowel disease/canine chronic enteropathy in dogs later in life (19–22).

NPMDs are raw non-processed meat-based diets consisting mainly of raw meats, raw organs, raw meaty bones, raw fish, raw eggs etc. NPMDs are high in protein and fats with low carbohydrate content. Additionally, the NPMDs contain raw vegetables, fruits, and berries. According to the average calculated from the NPMDs available in the Finnish market, it consists of 43.7% crude protein, 44% fat, 4.8% total carbohydrates, and 7.5% ash on dry matter basis (unpublished data). The UPCDs are commercial dry dog foods (kibbles) that contain a high amount of ultra-processed carbohydrates such as cereal grains (e.g., wheat, corn, oats, barley, rice, etc.) or potato starch, with a relatively low amount of animal-derived proteins when compared to the amount of fresh meat and bones included in the NPMDs. In addition to processed animal derived protein sources, the UPCD often contains processed plant-based proteins. The average composition of the UPCDs in Finland is as follows: 28.1% crude protein, 15.5% fat, 50% total carbohydrates, and 6.4% ash on a dry matter basis (unpublished data).

Maternal diet during pregnancy and postnatal diet both have a programming effect on the fetal immune system during these critical periods of developmental plasticity, thus influencing the long-term health of the offspring (47). Moreover, the dog microbiome’s sensibility to environmental exposures including the diet is time-dependent, where it is more sentient earlier in life and sensibility declines as the dog ages (48). Hence, this relatively short yet critical time-period from conception to 6 months of age is important for developing risk or protection.

The ontogeny of the immune system in dogs begins *in utero* (48–50) and is primarily reliant on appropriate nutrition (51, 52). One of the proposed mechanisms underlying fetal programming by diet is the direct impact of the maternal diet on fetal cytogenesis and organogenesis (17). The entire fetal growth and embryonic organ development, including the thymus, are mainly dependent on nutrients received from the mother (18). This can, in turn, result in permanent developmental changes in organs, tissues, and consequently physiological functions in the future (53). Therefore, providing pregnant dams with a species-appropriate diet such as NPMD is very crucial (54–56). The NPMD is a high-protein, high-fat diet, that secures proper protein and fat quantity and quality, as well as energy requirements that are important for immune system integrity. It has been reported that sufficient protein during pregnancy provides an ideal medium for the fetal thymus development and hence sustains better immune competence later in life (18). On the contrary, UPCD often lacks good quality animal-derived proteins, and instead has a high carbohydrate content, which is not an essential nutrient for dogs (57–59). A study on pregnant rats showed that there was a massive reduction in the level of the thymus and spleen proliferation in offspring exposed to a diet with a relatively low protein content compared to those exposed to a diet with adequate protein (60). A relatively low protein diet during pregnancy was also associated with several detrimental effects in mature rodent offspring, including impaired immunity and reduced density of cerebral cortex capillaries (61).

Early life environmental factors including pre- and postnatal diet, and the microbial composition, establish epigenetic changes that can affect developmental programming (16). Evidence suggests that the epigenetic changes resulting from early diet and microbiome interaction can be trans-generationally inherited, therefore having a substantial effect on evolution and an individual’s long-term health outcomes through modulating the immune response and the inflammatory molecular pathways (16, 62). This advocates for the importance of the early microbiome in driving the gut functions of the newborn for the rest of its life (51) and programming the immune system (63). The prenatal intestinal colonization is mainly shaped by the maternal gut, placental, and amniotic fluid microbiome (51, 64–66). A recent study reviewed the maternal diet-related changes in the immunity and microbiome of the offspring, both in humans and animals (52). Mirpuri reported that the maternal diet can alter the maternal microbiome, and this results in alternation in the offspring colonization either *in utero* and/or by vertical transmission via skin or the vaginal canal at birth. The maternal diet can also modify dietary metabolites and other dietary Toll-like receptors (TLR), which the fetus then will be exposed to in the uterus (52). Furthermore, the maternal diet has been found to alter cytokines, immunoglobulins, and other microbial products which also can alter TLR signaling in the embryonic gut and accordingly modulate the innate and adaptive newborn’s immune system (52, 67). Additionally, the presence of maternal diet-mediated cytokines in the amniotic fluid together with other growth factors have been found to stimulate the fetal gut immunity (68). However, transit dysbiosis in the maternal intestinal and intrauterine microbiota during pregnancy was correlated with metabolic and immune-mediated disorders in human offspring (69). Other pathways through which the maternal diet can mediate epigenetic modifications are via physiologic and metabolic changes that are accompanied by increased or decreased predisposition to the later development of diseases (70).

Owing to the above, the appropriate selection of the maternal diet during pregnancy has a profound effect on the health and immunity of the offspring. Many studies have revealed that dogs fed NPMD had a difference in the composition of the gut microbiome and metabolism versus those fed UPCD, where they found that dogs fed a NPMD showed a high gut and fecal microbial diversity compared to those fed dry kibbles (71–74). Schmidt et al. (75) reported that dogs fed a BARF (=Biologically Appropriate Raw Food, similar to the NPMD in the current study) diet showed a higher diversity of *Lactobacillales*, *Fusobacterium*, *Enterobacteriaceae*, and *Clostridium* while dogs fed an ultra-processed commercial diet (=kibble) showed a higher abundance of *Clostridiaceae, Ruminococcaceae, Erysipelotrichaceae,* and *Lachnospiraceae*. By contrast, the consumption of high-carbohydrate diets has been found to increase gut dysbiosis, inflammation, and gut permeability in mouse and calve studies (76–78). Interestingly, human studies have found that a leaky gut caused by gut dysbiosis can generate a chronic systemic inflammatory state, which can extend to extra-intestinal organs such as the skin and ears (79–81). Moreover, a study in humans reported that gut microbial dysbiosis was associated with loss of hearing as it stimulated cochlear inflammation (79). Another human study from Finland stated that gut microbiota diversity was inversely associated with the severity of atopic eczema in infants at 6 months of age (82). According to studies, dogs are expected to have the same associations as in humans and other mammals between the gut microbiome and the skin immune defense (43, 81). In addition, in a previous study we have demonstrated that the hereditary basis of a disease can be modified by environmental factors like the diet (83). Anturaniemi et al. (83) found that the gene transcription profile of the raw fed atopic dogs is compatible with an improvement of the innate immunity and reduction in the oxidative stress that can prevent hypersensitivities and disturbed immunity.

The role of the early diet on programming the immune system is not restricted to the maternal diet, also the postnatal diet has an effect (16, 17, 63). The immune system maturation in dogs begins at birth and becomes fully mature approximately at 6 months of age (48). The postnatal diet has a role in shaping the postnatal gut microbiome, and this results in epigenetic signatures that can act on the properties of the gut mucosal barriers and their defensive role in opposition to later insults, therefore possibly prompting or restraining the later development of inflammatory diseases (16, 63). The postnatal diet provides the required nutrients for the newborns’ growth and for their organ development (18). Besides the benefits and risks of the NPMD and UPCD mentioned above, a study reported that adequate high quality protein intake is essential for the proliferation of gut mucosal goblet lymphocytes, which have a role in eliminating infections that can generate disease (84). Moreover, a human study found that there was a positive association between inadequate protein intake and an increased risk of different diseases in children (85). Furthermore, some nutritional factors have been found to be partially responsible for hearing loss in humans, where they found that a higher intake of carbohydrates and a lower intake of protein was associated with bad hearing status (86).

Another modifiable domestic risk factor that was observed in the current study is the question of being born in the same family that the dog now lives with, or not. We found that dogs that were born and continued to live in the same family were exposed to a lower risk to develop otitis later in life, while having been born in a different family, was associated with an increased 2.2-fold risk. These findings agree with our previous findings, where we found the same associations with CAD (19, 24). Research showed that daily in-house contact between the dog, its dam and siblings, home environment, and also between the puppy and its owner, reflected immune system adaptation to the same environmental stimuli through sharing the same microbiome (87–89). An opposing theory also exists: Garrigues et al. (51) found that puppies moving into big cities after leaving the birth kennel, showed a higher bacterial diversity compared to dogs living in small cities. This might be due to the exposure to a wide range of environmental influences and microbial exchange with other dogs and people during leash walking which means that the microbial development is affected by the geographic localization (51, 90).

An important environmental risk factor in the current study was that dogs that were exposed to sunlight for at least 1 hour daily during their early postnatal life (= from 1 to 2 months of age) showed a lower risk of developing otitis later in life versus dogs that were totally deprived of sunlight exposure. This result is similar to our previous findings in dogs suffering from CAD (19). The same has been observed in early childhood, where regardless of vitamin D status, they found that the exposure to direct ultraviolet rays decreased AD development in young children (91). Additionally, when a dog is outdoors for an hour or more daily, it also guarantees exposure to different environmental allergens. This, in turn, will stimulate the dogs’ immune system (51).

The type of flooring the dogs were brought up on during puppyhood and up to 6 months of age was also associated with otitis later in life, although it did not reach significance (*p*=0.051). We found that dogs that had been raised on a dirt floor (earth) or lawn had a 0.7-fold lower incidence of otitis later versus dogs using other floor types. Also, this is akin to our previous findings (19, 21). The positive role of soil microbiome on the immune system stimulation is well accepted and has been elucidated in several studies (20, 92).

Our analysis Figure 4 shows that the prevalence of otitis was significantly higher within dogs that were not consuming NPMD at all during puppyhood, while the prevalence of otitis was significantly reduced within the groups of dogs that were consuming from 26 to 50%, 51–75%, and from 76 to 100% of their diet as non-processed foods. On the other hand, eating 76–100% of the diet during puppyhood as kibble significantly increased the prevalence of otitis later in their life. These findings agree with our previous findings regarding AASS (21), where we found that consumption of at least 20% of a NPMD reduced the prevalence of AASS for dogs in their later life, while the consumption of 80% or more of UPCD of the dog’s diet significantly increased the prevalence of AASS later in life. This implies that consuming only a small ratio of the diet as NPMD (less than 26%) might not be enough to exert a beneficial impact on the immune system, whereas supplying the puppy with a sufficient quantity of NPMD provides the required nutrients from high-quality sources, as discussed above.

## Strengths and limitations

The main strength of the current study is that we have tested a wide range of heterogeneous variables over four early-life periods starting from conception up to puppyhood. The research provides an epidemiological paradigm that also can be used in human research. Moreover, the study accounted for reverse causality by setting cut points for the age of the cases and the controls included in the study, based on a general age of onset (Figure 1). Another strength is that the data from the FFQ has been validated, securing its reliability (23).

The current study also has some limitations. Our measure of otitis in dogs is based on owners’ reports and not on a veterinarian’s confirmed clinical diagnosis. However, we handled this weakness by posing a set of additional questions related to the targeted disease, otitis. For instance, we asked how often the dog had suffered from otitis? At which age the dog suffered from otitis for the first time? If the dog still suffers from otitis or not? etc. These related questions helped to validate the owners’ responses. The owners’ responses were also confirmed by sending the owners emails to re-answer the survey (manuscript under preparation). As the study was cross-sectional, this might have led to recall bias. However, in addition to the data validation, the questions used in the FFQ were multiple-choice questions, helping to overcome the recall bias.

## Conclusion

In conclusion, the current study showed that the early life diet and some environmental exposures were associated significantly with the incidence of otitis in dogs over 1 year of age. We conclude that the consumption of a NPMD during pregnancy, early and late postnatal life, was associated with a reduced risk of otitis at over 1 year of age. On the contrary, the consumption of an UPCD during the same periods was associated with a higher risk of otitis development later in life. The study recommended that the consumption of NPMD should be >25% of the dog’s whole diet while the consumption of UPCD should be <75% of the dog’s diet. Moreover, being born in the current family was associated with a lower incidence of otitis at over 1 year of age when compared to those puppies that moved to new families. Also, daily sunlight exposure for at least 1 hour was associated with a lower incidence of otitis in dogs at over 1 year of age compared to subjects not exposed to sunlight at all. Furthermore, the current study identified a lower risk for otitis development in dogs raised on a dirt floor or lawn during late puppyhood versus dogs raised on other kinds of floors. The study also identified the maternal history of otitis, CAD, and dog breed as significant risk factors for otitis development.

This study provides new insights into otitis in dogs that can inspire researchers and veterinarians to apply a primary preventive strategy for otitis in dogs. These findings suggest causality but do not prove it. Diet interventions should be conducted to confirm our observations, with a special focus on early diets.

## Data availability statement

The raw data supporting the conclusions of this article will be made available by the authors, without undue reservation.

## Ethics statement

The studies involving animals were reviewed and approved by the Ethical Board of Viikki Campus, University of Helsinki (29.4.2016). Written informed consent from the owners was not required to participate in this study in accordance with the national legislation and the institutional requirements.

## Author contributions

MH and AH-B planned, designed, drafted the study, performed the data extraction, and did the statistical analysis. AH-B organized the database. MH, KV, NB, RM, SR, JA, AE-L, and AH-B wrote sections of the manuscript and edited it. All authors contributed to the article and approved the submitted version.

## Footnotes

^1^ https://www.fci.be/
