# Supplementary material for: Early life programming by diet can play a role in risk reduction of otitis in dogs
Source: Front Vet Sci. 2023 Nov 6;10:1186131. doi: 10.3389/fvets.2023.1186131 (PMC10657834; doi:10.3389/fvets.2023.1186131)
Supplement: Supplementary file 1 [file Table_1.docx]

**Table S1. Canine otitis-prone breeds based on other authors’ observations.**

| **Authors/Source** | **Otitis prone breeds** |
| --- | --- |
| ***O´Neill et al. 2021*** | Basset Hound, Chinese Shar Pei, Labradoodle, Beagle, Golden Retriever, Cockapoo, American Bulldog, French Bulldog, English Bulldog, Pug, Cavapoo, West Highland White Terrier, Rottweiler, Cocker Spaniel, Labrador Retriever, Bichon Frise. |
| ***Dodds. 2011*** | Basset Hound, Chinese Shar Pei, English Cocker Spaniel, English Springer Spaniel, German Shepherd, Miniature poodle, Old English sheepdog, Toy poodle, Wire-haired pointing griffon, American cocker spaniel |
| ***Nødtvedt et al. 2006*** | West Highland White Terrier, Boxer, German Shepherd Dog, Bull Terrier, Labrador Retriever, American Staffordshire Terrier, Dalmatian, Rhodesian Ridgeback, Staffordshire Bull Terrier, Newfoundland, Danish-Swedish Farmdog, Irish Soft Coated Wheaten Terrier, Welsh Springer Spaniel, Welsh Terrier |
