# Supplementary material for: Early life programming by diet can play a role in risk reduction of otitis in dogs
Source: Front Vet Sci. 2023 Nov 6;10:1186131. doi: 10.3389/fvets.2023.1186131 (PMC10657834; doi:10.3389/fvets.2023.1186131)
Supplement: Supplementary file 2 [file Table_2.docx]

| Table S2. Characteristics of the study population and covariates. | | | | | | | |
| --- | --- | --- | --- | --- | --- | --- | --- |
| N. | | Covariates | Categories | Canine Otitis %(n) | | | |
|  |  |  |  | **Cases, %**  **(n=1237)** | **Controls, %**  **(n=1827)** | **Total, %**  **(n=3064)** | |
| 1 | | Maternal history of Otitis | Mothers without otitis | 60.9 (262_a_) | 93.2 (806_b_) | 82.5 (1068) | |
|  |  |  | Mothers with otitis | 39.1 (168_a_) | 6.8 (59_b_) | 17.5 (227) | |
| 2 | | Canine AASS | Dogs without AASS | 64.5 (743_a_) | 87.3 (1558_b_) | 78.4 (2301) | |
|  |  |  | Dogs with AASS | 35.5 (409_a_) | 12.7 (226_b_) | 21.6 (635) | |
| 3 | | Dog breed | Non-otitis prone breeds | 33.7 (333_a_) | 56.0 (756_b_) | 46.5 (1089) | |
|  |  |  | Otitis prone breeds | 66.3 (656_a_) | 44.0 (595_b_) | 53.5 (1251) | |
| 4 | | Dog sex | Male | 48.8 (588_a_) | 43.8 (778_b_) | 45.8(1366) | |
|  |  |  | Female | 51.2 (616_a_) | 56.2 (998_b_) | 54.2 (1614) | |
| 5 | | Dog color | White >50% | 20.8 (240_a_) | 19.5 (336_a_) | 20.0 (576) | |
|  |  |  | White <50% | 79.2 (916_a_) | 80.5 (1383_a_) | 80.0 (2299) | |
| 6 | | Dog’s ear shape | Erect ears | 23.4 (236_a_) | 40.9 (566_b_) | 33.5 (802) | |
|  |  |  | Semi-erect ears | 13.9 (140_a_) | 18.1 (250_b_) | 16.3 (390) | |
|  |  |  | Dropped ears | 62.7 (633_a_) | 41.1 (569_b_) | 50.2 (1202) | |
| 7 | | Hairy/pilose ears | Non-hairy/pilose ears | 82.3 (830_a_) | 85.8 (1188_b_) | 84.3 (2018) | |
|  |  |  | Hairy/pilose ears | 17.7 (179_a_) | 14.2 (197_b_) | 15.7 (376) | |
| 8 | | Mother’s diet during pregnancy | NPMB | 5.6 (41_a_) | 8.9 (103_b_) | 7.6 (144) | |
|  |  |  | UPCD | 94.4 (694_a_) | 91.1 (1058_b_) | 92.4 (1752) | |
| 9 | | Was the mother dewormed during pregnancy? | Yes | 97.5 (696_a_) | 97.2 (1131_a_) | 97.3 (1827) | |
|  |  |  | No | 2.5 (18_a_) | 2.8 (33_a_) | 2.7 (51) | |
| 10 | | Was mother vaccinated during pregnancy? | Yes | 52.8 (211_a_) | 49.4 (337_a_) | 50.6 (548) | |
|  |  |  | No | 47.3 (189_a_) | 50.6 (345_a_) | 49.4 (534) | |
| 11 | Age / years, mean ± SD^1^ | | | 5.25 ± 2.94 | 5.56 ± 2.70 | 5.44 ± 2.8 |  |
| 12 | | Mother’s diet during lactation | NPMB | 6.5 (45_a_) | 8.4 (93_a_) | 7.7 (138) | |
|  |  |  | UPCD | 93.5 (646_a_) | 91.6 (1014_a_) | 92.3 (1660) | |
| 13 | | Season of birth | Winter (Dec-Feb) | 27.8 (337_a_) | 24.2 (437_b_) | 25.7 (774) | |
|  |  |  | Spring (March-May) | 31.7 (384_a_) | 32.5 (586_a_) | 32.2 (970) | |
|  |  |  | Summer (June-Aug) | 21.4 (259_a_) | 23.6 (425_a_) | 22.7 (684) | |
|  |  |  | Autumn (Sept-Nov) | 19.2 (233_a_) | 19.7 (355_a_) | 19.5 (588) | |
| 14 | | Puppy’s first solid diet | NPMD | 6.3 (46_a_) | 8.6 (96_a_) | 7.7 (142) | |
|  |  |  | UPCD | 93.7 (687_a_) | 91.4 (1024_a_) | 92.3 (1711) | |
| 15 | | Frequency of outdoor activity | Many times / day | 53.2 (488_a_) | 61.2 (857_b_) | 58.0 (1345) | |
|  |  |  | Once / day | 17.0 (156_a_) | 15.7 (220_a_) | 16.2 (376) | |
|  |  |  | A few times / week | 14.1 (129_a_) | 11.2 (157_a_) | 12.3 (286) | |
|  |  |  | A few times / month | 6.2 (57_a_) | 3.9 (55_b_) | 4.8 (112) | |
|  |  |  | Not at all | 9.5 (87_a_) | 8.0 (112_a_) | 8.6 (199) | |
| 16 | | Sunlight exposure, hours / day | Not at all | 53.8 (348_a_) | 44.8 (459_b_) | 48.3 (807) | |
|  |  |  | ≥ 1 hour | 46.2 (299_a_) | 55.2 (566_b_) | 51.7 (865) | |
| 17 | | Type of flooring | Dirt / lawn floor | 5.7 (55_a_) | 7.6 (111_a_) | 6.9 (166) | |
|  |  |  | Non-dirt / lawn floor | 94.3 (908_a_) | 92.4 (1346_a_) | 93.1 (2254) | |
| 18 | | Body condition score | Overweight puppies | 14.0 (145_a_) | 14.8 (228_a_) | 14.5 (373) | |
|  |  |  | Normal weight puppies | 75.5 (781_a_) | 76.5 (1175_a_) | 76.1 (1956) | |
|  |  |  | Underweight puppies | 10.4 (108_a_) | 8.7 (133_a_) | 9.4 (241) | |
| 19 | | Puppy diet | NPMD | 7.2 (44_a_) | 11.0 (95_b_) | 9.4 (139) | |
|  |  |  | UPCD | 92.8 (566_a_) | 89.0 (766_b_) | 90.6 (1332) | |
| 20 | | Was the dog born into the same human family as where it stayed as adult? | No | 95.6 (1183_a_) | 90.4 (1652_b_) | 92.5 (2835) | |
|  |  |  | Yes | 4.4 (54_a_) | 9.6 (175_b_) | 7.5 (229) | |
| 21 | | Outdoor activity, hours / day | < 0.5 | 2.2 (23_a_) | 2.4 (36_a_) | 2.3 (59) | |
|  |  |  | 0.5 – 1.0 | 28.0 (293_a_) | 26.5 (401_a_) | 27.1 (694) | |
|  |  |  | 1.0 – 2.0 | 53.8 (562_a_) | 51.2 (776_a_) | 52.3 (1338) | |
|  |  |  | > 2.0 | 16.0 (167_a_) | 19.9 (302_b_) | 18.3 (469) | |
| 22 | | Sunlight exposure, hours / day | ≤ 1 | 27.6 (268_a_) | 25.4 (348_a_) | 26.3 (616) | |
|  |  |  | > 1 | 72.4 (703_a_) | 74.6 (1023_a_) | 73.7 (1726) | |
| 23 | | Type of flooring | Dirt / lawn | 8.9 (98_a_) | 12.2 (223_b_) | 11.0 (321) | |
|  |  |  | Non-dirt / lawn | 91.1 (1002_a_) | 87.8 (1604_b_) | 89.0 (2606) | |
| 24 | | Body condition score | Overweight puppies | 7.2 (78_a_) | 6.0 (95_a_) | 6.5 (173) | |
|  |  |  | Normal weight puppies | 65.7 (714_a_) | 69.3 (1099_a_) | 67.9 (1813) | |
|  |  |  | Underweight puppies | 27.1 (294_a_) | 24.7 (391_a_) | 25.6 (685) | |
| 25 | | Was the puppy vaccinated 2-4 times under 1 year of age? | Yes | 99.2 (1210_a_) | 99.0 (1776_a_) | 99.1 (2986) | |
|  |  |  | No | 0.8 (10_a_) | 1.0 (18_a_) | 0.9 (28) | |
| 26 | | Was the puppy dewormed 2-10 times under 1 year of age? | Yes | 99.6 (1200_a_) | 99.0 (1744_a_) | 99.3 (2944) | |
|  |  |  | No | 0.4 (5_a_) | 1.0 (17_a_) | 0.7 (22) | |

^1^ Age / years calculated as mean ± SD.

_a,b_ Different letters means significant difference within the same row at p<0.05.
